# Measuring and Sorting Cell Populations Expressing Isospectral Fluorescent Proteins with Different Fluorescence Lifetimes

**DOI:** 10.1371/journal.pone.0109940

**Published:** 2014-10-10

**Authors:** Bryan Sands, Patrick Jenkins, William J. Peria, Mark Naivar, Jessica P. Houston, Roger Brent

**Affiliations:** 1 Division of Basic Sciences, Fred Hutchinson Cancer Research Center, Seattle, Washington, United States of America; 2 Department of Chemical Engineering, New Mexico State University, Las Cruces, New Mexico, United States of America; 3 Darkling X, LLC, Los Alamos, New Mexico, United States of America; University of Birmingham, United Kingdom

## Abstract

Study of signal transduction in live cells benefits from the ability to visualize and quantify light emitted by fluorescent proteins (XFPs) fused to different signaling proteins. However, because cell signaling proteins are often present in small numbers, and because the XFPs themselves are poor fluorophores, the amount of emitted light, and the observable signal in these studies, is often small. An XFP's fluorescence lifetime contains additional information about the immediate environment of the fluorophore that can augment the information from its weak light signal. Here, we constructed and expressed in *Saccharomyces cerevisiae* variants of Teal Fluorescent Protein (TFP) and Citrine that were isospectral but had shorter fluorescence lifetimes, ∼1.5 ns vs ∼3 ns. We modified microscopic and flow cytometric instruments to measure fluorescence lifetimes in live cells. We developed digital hardware and a measure of lifetime called a “pseudophasor” that we could compute quickly enough to permit sorting by lifetime in flow. We used these abilities to sort mixtures of cells expressing TFP and the short-lifetime TFP variant into subpopulations that were respectively 97% and 94% pure. This work demonstrates the feasibility of using information about fluorescence lifetime to help quantify cell signaling in living cells at the high throughput provided by flow cytometry. Moreover, it demonstrates the feasibility of isolating and recovering subpopulations of cells with different XFP lifetimes for subsequent experimentation.

## Introduction

Understanding the quantitative function of cell signaling systems requires measurements of the molecules and reactions by which they operate. In some studies, investigators use antibodies to assay activation of signaling proteins in fixed, permeabilized cells [1]–[4]. Even with very high quality antibodies, such measurements can be inaccurate, due in part to a tradeoff between complete permeabilization and complete fixation [5]. Moreover, work with dead, fixed cells by definition cannot track signaling function in the same cells over time. For these reasons, some quantitative cell signaling research requires real-time measurements in live cells [6]. Such studies measure the operation of signaling systems by quantifying the molecular events; for example, protein re-localization, oligomerization, or activation of protein kinases [6]–[7].

Currently, quantification of signaling in living cells relies on acquisition, by microscopy, of light at different wavelengths emitted from genetically encoded fluorescent reporter proteins. These proteins are often chimeras comprised of proteins or parts of proteins involved in signaling fused to derivatives of *Aquoera victoria* Green Fluorescent Protein or other fluorescent proteins here called XFPs (see comprehensive review by [8]). Examples of cell signaling events quantified by XFP-containing reporters include relocalization of scaffold proteins to the inside of the cell membrane [9]–[10], and of protein kinases and transcription factors to the nucleus [11]–[12]. They include association and dissociation of members of protein complexes, measured by gain and loss of Foerster Resonance Energy Transfer (FRET) between a “donor” XFP and longer wavelength “acceptor” XFP, when those XFPs are fused to different complex members [9], [13–15). They include activation of specially designed biosensors [16], in which enzymatic activity, changes in protein conformation, and changes in FRET are used to quantify a variety of biochemical processes including GTPase activity [17]–[19], and protein kinase activity [7], [20]–[25].

Quantification that depends on fluorescent reporter proteins must overcome the fact that XFPs are poor fluorophores. Compared to chemical fluorophores such as rhodamine dyes, fluoresceins, or quantum dots [26]–[29], XFPs have low quantum yields, are prone to photobleaching, and have broad emission spectra which limit the number of spectrally distinguishable colors researchers can engineer a cell to emit [8], [30]. Some studies use chimeric XFP reporter proteins that replace native cell signaling proteins present in small numbers, i.e. less than 100 s–1000 s of copies per cell [31], and thus produce weak fluorescent signals. Moreover, cells have background autofluorescence [32], considered in [33]–[34]. For these reasons, the signals above background from XFPs that researchers use to quantify signaling in living cells are often weak.

When using microscopy to image XFPs in signaling studies, the investigator can compensate for low fluorescent signal by exciting the cells and collecting signal for longer times, limited only by the eventual photobleaching of the XFPs. However, when using flow cytometry [35], the investigator can acquire XFP signal only during the time the cell passes through the laser beam (typically, microseconds), but to some extent can compensate for the short signal acquisition time by the brighter excitation light provided by the cytometer's lasers.

In addition to measurement of fluorophore's fluorescence intensity within a specified wavelength range, it is also possible to measure its fluorescence lifetime. This is the mean time between the fluorophore's excitation and its decay to the ground state [36], typically several nanoseconds. This lifetime is comprised of a natural “radiative lifetime,” characteristic of each species of fluorophore, and a contribution brought about by the fluorophore's environment. For example, a crowded atomic environment near the fluorophore shortens the lifetime by providing more paths for non-radiative decay from the excited state [36]. The time that it takes an excited fluorophore of a known species to emit a photon thus contains information about the fluorophore's immediate cellular environment.

Information from fluorescence lifetime measurements can complement information from measurements of fluorescence intensity. For example, FRET occurring during the association of a donor and acceptor XFP pair causes a decrease in the ratio of donor-to-acceptor fluorescence, and a concomitant decrease in the fluorescence lifetime of the donor [14]. The lifetime shift measurement thus adds to the information provided by the intensity ratio measurement. In the future, we also hope that fluorescence lifetime information might increase the number of distinguishable XFP signals from individual cells, facilitating the use of Bayesian network methods [37] in live cells to find features of signaling networks specific to different disease states and generate hypotheses about cause and effect relationships among measured variables [38]–[40].

One way to measure fluorescence lifetime is by “frequency domain” methods [41], in which the investigator excites collections of fluorophores using light modulated sinusoidally at radio frequencies (RF, here, 1–50 MHz). The excited fluorophores emit light modulated at the same frequency as the excitatory light, but the modulation is delayed in phase and reduced in modulation depth (ratio of the modulation amplitude to the average intensity, see e.g. [42]. The investigator then determines lifetime by using the phase delay of the emitted light relative to the excitatory light (larger phase delays mean longer lifetimes), and/or by using the demodulation (the ratio of the emission modulation depth to the illumination modulation depth, smaller ratios mean longer lifetimes)[14]. Simultaneous measurement of both phase delay and demodulation in frequency domain fluorescence lifetime measurements enables the use of “phasor analysis” [51, Appendix A]. In this, the investigator uses the phase and demodulation measurements to construct phasors– complex numbers with magnitudes equal to the measured demodulation factors, and arguments equal to the measured phase delays. The investigator plots these as points in the complex plane, so that each point's distance from the origin is proportional to the phasor's demodulation factor, and the line from the origin to the point forms an angle with the real axis equal to the phasor's phase delay. Such phasor plots can reveal the presence of mixtures of fluorophores with different lifetimes, or of fluorophores with multiple decay paths, and can help troubleshoot instruments during their development (see Methods).

During the 1990s, two groups developed flow cytometers that measured fluorescence lifetime [43]–[44]. Both made a series of analog hardware additions to the signal detectors. Each mixed inputs of the same frequency (i.e., used homodyning), and then carried out low-pass filtering and an analog division. One group (Steinkamp and coworkers) first split the output of the fluorescence detector into two identical signals [44]. They mixed (analog multiplied) each signal with a different reference signal. The reference signals were separated from each other in phase by 90 degrees. By low-pass filtering these mixtures, they obtained two pulses which each corresponded to the envelope of the fluorescence signal, with heights that indicated the alignment in phase (correlation) between each reference signal and the fluorescence signal. They took the ratio of these two pulses to obtain the fluorescence lifetime directly. The other group (Pinsky and coworkers), used similar techniques, but used scattered light for their only reference signal, and measured the cosine of the lifetime-associated phase shift rather than the lifetime [43]. By these analog homodyning methods, both groups measured lifetimes in mixed subpopulations of cells and beads labeled with non-protein fluorophores (fluorescent dyes or quantum dots) of different lifetimes [43]–[46].

Recently, using the analog methods of Steinkamp et al., we measured fluorescence lifetime from fluorescent microspheres and from fixed, stained cells [47]. Moreover, we demonstrated the ability to sort mixed populations of fluorescent beads with different lifetimes and cells stained with ethidium bromide and propidium iodide (which have different lifetimes) to ∼90% purity [47]. In parallel, we developed digital means to quantify phase shift in flow [48]. To do so, we developed fast, dedicated digital signal data acquisition systems [49], [50] that used analog to digital converters (ADCs) to capture waveforms, and a Field Programmable Gate Array (FPGA) to calculate FFTs from which we derived fluorescence lifetimes from differences in phase [49] and that provided analog outputs that interfaced with a commercial cell sorter [49]. We used this digital system to measure fluorescence lifetime of microspheres and fixed, stained cells and, to sort a mixture of microspheres with 2 ns and 7 ns fluorescence lifetimes to 98% purity [48].

Here, we constructed *Saccharomyces cerevisiae* that expressed fluorescent protein variants designed to have identical emission spectra (i.e., ″is spectral) but different fluorescence lifetimes (∼1.5 ns and ∼3 ns). To quantify these lifetimes, we used modified frequency-domain microscopic and flow cytometric instruments. For flow cytometry, we acquired data digitally from cells on an improved digital system with faster ADCs and a faster FPGA, and used it to generate complex numbers we called “pseudophasors” from information about phase and amplitude. Plots of pseudophasors in the complex plane facilitated identification and “gating” of desired cell populations. We developed algorithms that computed pseudophasors on this digital hardware very rapidly, fast enough to permit their use to sort on small (1.5 ns) differences in lifetimes for hundreds of cells per second. We then used these capabilities to measure fluorescence lifetimes of isospectral live cell subpopulations, and to sort mixed populations into nearly pure populations we scored phenotypically by colorimetric assay of colonies descended from the sorted cells. The work opens the way to acquire fluorescence lifetime information from genetically encoded, fluorescent protein reporters in live cells at the high throughput provided by flow cytometry, and to sort and recover subpopulations of live cells with different lifetimes for further genetic and molecular analysis.

## Results

### Construction of cells expressing isospectral XFPs with altered lifetimes

**Figure 1:**
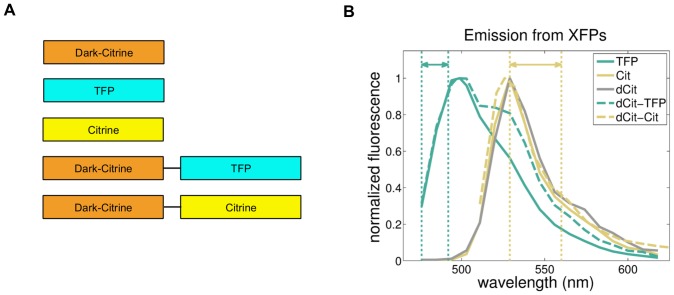
Fluorescent proteins. A) Body plan of fluorescent proteins. DarkCitrine is fused to the N terminus of TFP and Citrine with a GSGG linker (black bar). B) Emission spectra from yeast expressing each XFP excited by a 458 nm laser and emission collected from 460–650 nm. (Double-headed arrows indicate wavelength bands used in Figure 2)

We designed and constructed pairs of proteins with identical emission spectra (*isospectral*) but different fluorescence lifetimes (*allothoric*). We used standard methods [52] to construct genes that encoded monomeric, pH desensitized, yeast-codon- optimized versions of four different XFPs: ymTFP1 [53], here called “TFP”), ymECitrineF46L (based on the original protein in [54], and here called “Citrine” or “Cit”), and two different chimeric proteins that fused TFP and Citrine to a variant of Citrine based on the sREACh derivative of YFP [55], here called “dark Citrine” and “dCit”). We connected TFP and Citrine to the C terminus of dCit via a GSGG linker. We believed that, due to FRET to the dCit acceptor, the TFP and Citrine moieties in these chimeras would have shorter fluorescence lifetimes. Figure 1A shows the structure of these proteins.

We made yeast strains expressing the fluorescent proteins by introducing the above fusion genes into the high copy number yeast expression plasmid pADNS (2u, LEU2+) [56], which directs the synthesis of proteins encoded by downstream sequences under the control of the ADH1 promoter [57], and introducing these plasmids, along with an empty vector control plasmid, into the haploid S288C, BY4741 [58]. Yeast strains used in this work are listed in Table 1.

**Table 1:** Yeast Strains.

Strain	Parent	Plasmid^a^^,^ b	Genotype
BSY003	BY4741	pBS3/P_ADH1_-TFP	MATa ho his3Δ0 leu2Δ0 met15Δ0 ura3Δ0
BSY004	BY4741	pBS9/P_ADH1_-Citrine	MATa ho his3Δ0 leu2Δ0 met15Δ0 ura3Δ0
BSY008	BY4741	pADNS/P_ADH1_-empty	MATa ho his3Δ0 leu2Δ0 met15Δ0 ura3Δ0
BSY010	BY4741	pBS19/P_ADH1_-darkCitrine	MATa ho his3Δ0 leu2Δ0 met15Δ0 ura3Δ0
BSY014	BY4741	pBS24/P_ADH1_-dCit-TFP	MATa ho his3Δ0 leu2Δ0 met15Δ0 ura3Δ0
BSY015	BY4741	pBS32/P_ADH1_-dCit-Citrine	MATa ho his3Δ0 leu2Δ0 met15Δ0 ura3Δ0
BSY034	WY581	pBS3/P_ADH1_-TFP	MATa leu2Δ0 ADE2
BSY035	WY639	pBS24/P_ADH1_-dCit-TFP	MATa leu2Δ0 ade2::hisG

a All plasmids are 2 µm and Leu2 marked.

b plasmid name/XFP being expressed.

We checked the fluorescence of each strain by microscopy on a wide field fluorescence microscope with excitation by an LED light source at 462 nm (TFP constructs) or 514 nm (citrine constructs). As expected, yeast expressing dCit-TFP and dCit-Citrine were dimmer than strains expressing TFP and Citrine. Cells from all strains showed significant heterogeneity in fluorescence intensity, which we attributed to cell-to-cell variation in the copy number of the 2 µm plasmids that directed the synthesis of the XFPs.

We measured the emission spectra of the XFP-expressing strains using the spectral scan function on an LSM780 confocal microscope with excitation by a 458 nm argon laser line and emission collection range of 460–650 nm (Figure 1B). Citrine and dCit-Citrine had identical spectra that match published data [54]. We therefore refer to them as isospectral. By contrast TFP and dCit-TFP emission matched published data at peak wavelengths [53], i.e. they had identical emission spectra there, but at longer wavelengths dCit-TFP emitted more light, possibly due to excitation via FRET by the TFP donor. Since the fluorescence collection range on our microscope (468–492 nm) covered only the TFP peak emission, we considered these XFPs isospectral for the microscopy in this work.

### Measurement of altered lifetimes by microscopy

We grew and processed for imaging cells expressing different XFPs as described (Materials and Methods). We imaged these cells with two FLIM systems: a widefield system (FLIM-w), and a confocal system (FLIM-c). The configuration of FLIM-w permitted imaging of TFP and dCit-TFP cells but not Citrine and dCit-Citrine. The configuration of FLIM-c permitted measurements of all TFP and Citrine derivatives. For each system, we recorded sets of images (“phase stacks”) of the same field of cells at different relative phasing of the modulated camera gain with respect to the modulated illumination. From these phase stacks, we extracted fluorescence lifetime for all pixels within the field of view that corresponded to emission from sufficiently bright cells. We took the average lifetime of pixels corresponding to specific cells to generate lifetime values per cell.

**Figure 2:**
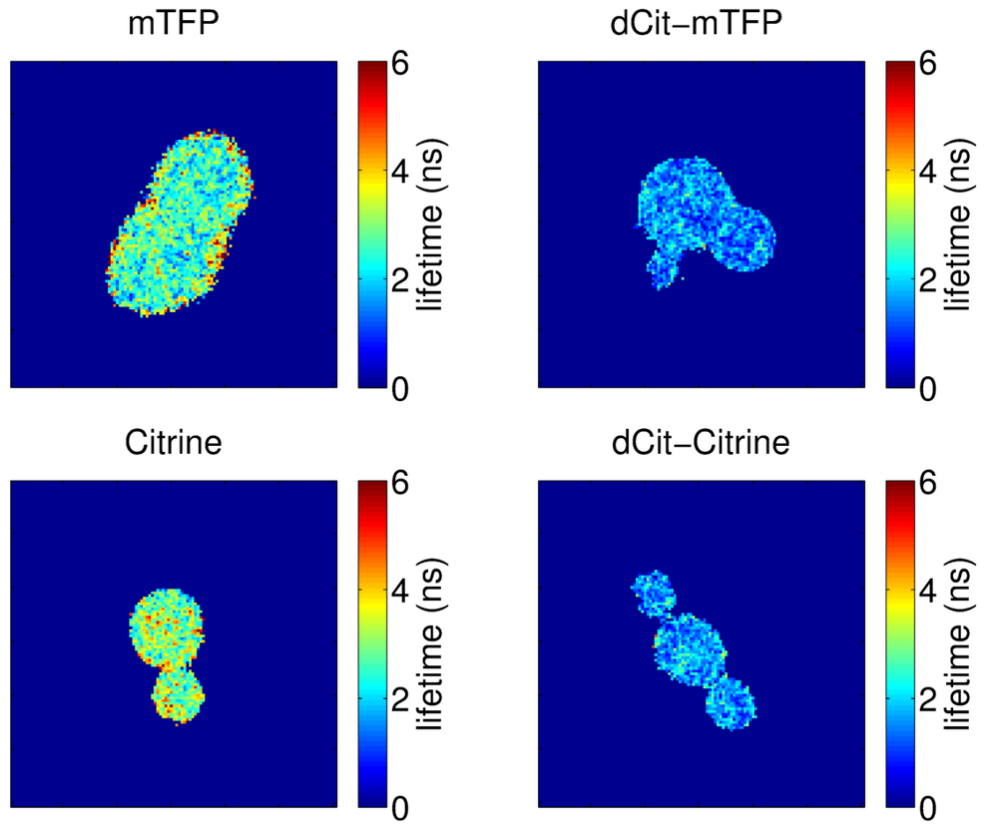
dCit-TFP and dCit-Citrine proteins have shorter lifetimes. False color fluorescence lifetime images of yeast cells expressing TFP (upper left), dCit-TFP (upper right), Citrine (lower left) and dCit-Citrine (lower right). Light from regions colored dark blue was below the threshold for lifetime computation.

Figure 2 shows representative false color “lifetime images” of cells from FLIM-c. Cells expressing dCit-TFP and dCit-Cit had visibly shorter lifetimes than cells expressing TFP and Citrine; below 2 ns compared to lifetimes above 3 ns. On FLIM-c and FLIM-w, pixels from cells expressing TFP had average lifetimes of 2.71 and 3.0 ns respectively, while pixels from cells expressing dCit-TFP had average lifetimes of 1.53 and 1.7 ns. As measured on FLIM-c, pixels from cells expressing Citrine had an average lifetime of 2.97 ns, while pixels from cells expressing dCit-Citrine had an average lifetime of 1.64 ns. Data for all microscopy experiments are listed in Table 2. Table 2 also shows the standard deviations in measured lifetimes of cell-associated pixels in the confocal slices in Figure 2. Within this measurement uncertainty, both TFP and Citrine, as measured on FLIM-c, had fluorescence that was consistent with simple monoexponential decays (see section on phasor analysis, below).

**Table 2:** Fluorescence lifetime by microscopy and flow cytometry.

XFP expressed	FLIM-c	FLIM-w	Cytometer
**None**	Not measurable	Not measurable	ND
**TFP**	2.7±1.1 ns	3.0 ns (phase)	2.85±0.54 ns
		2.9 ns (modulation)	
**dCit-TFP**	1.53±0.55 ns	1.7 ns (phase)	1.94 ±0.52 ns
		1.5 ns (modulation)	
**Citrine**	2.97±0.85 ns	ND	ND
**dCit-Citrine**	1.64±0.59 ns	ND	ND

ND = not done.

± = standard deviation for pixels within each confocal slice.

### Measurement and sorting of cell populations with different lifetimes in flow.

We devised means to measure XFP lifetimes in cells by flow cytometry and to sort cell populations based on XFP lifetime differences. To do so, we configured a FACSVantage SE flow cytometer (Becton Dickinson) to allow detection of TFP and dCit-TFP fluorescence and to capture digitized waveforms from PMTs collecting side-scattered light (the SSC channel) and fluorescence (the FL1 channel), see Figure S1 for a diagram of the instrument.

To carry out these cytometric experiments, we collected both fluorescence and scattered laser light from the yeast at 90° to the laser excitation. The excitation light, scattered light, and the fluorescence were all modulated at 25 MHz. At this frequency, each cell was illuminated by a few hundred complete modulation cycles during its passage through the excitation beam. The scattered laser light served as a reference for the phase shift of the fluorescence signal [43]. We used a custom-built data acquisition system to capture waveforms from the PMT signals, store them and send them to a PC. We used Kytos software (DarklingX LLC., Los Alamos, NM) to compute lifetime using the delay in phase between the collected waveforms, for each cell that triggered the detector.

We used the modified instrument to collect fluorescence and scattered light from TFP and dCit-TFP expressing strains and to compute lifetime from phase shift. Table 2 shows the results. In TFP-expressing cells, TFP had an average lifetime of 2.85 ±0.54 ns. In dCit-TFP-expressing cells, dCit-TFP had an average lifetime of 1.94 ± 0.52 ns. Both values are in good agreement with the average lifetime measurements determined by our microscope measurements.

We then used the modified flow cytometer to sort sub-populations by fluorescence lifetime. To do so, we used the same two waveforms, corresponding to excitatory and emitted light to generate pseudophasors for each passing cell. A plot of pseudophasors in the complex plane displays differences in lifetimes as differences in phase. Such a plot enables the investigator to easily select well-separated high-amplitude events for which the phase delay distributions do not overlap. We used these pseudophasor plots to draw polygons in the complex plane that defined sort gates (Figure S2).

We used this modified cytometer to sort cells that expressed TFP and cells that expressed dCit-TFP and that also carried an *ade2-* mutation. Yeast Cells with a wild type *ADE2+* gene form normal colored (off-white) colonies on adenine depleted medium, while cells that carry mutations in the gene (*ade2-*) accumulate a red pigment [59]. This phenotype allowed us to distinguish colonies of cells expressing TFP from colonies of cells expressing dCit-TFP by color.

**Figure 3:**
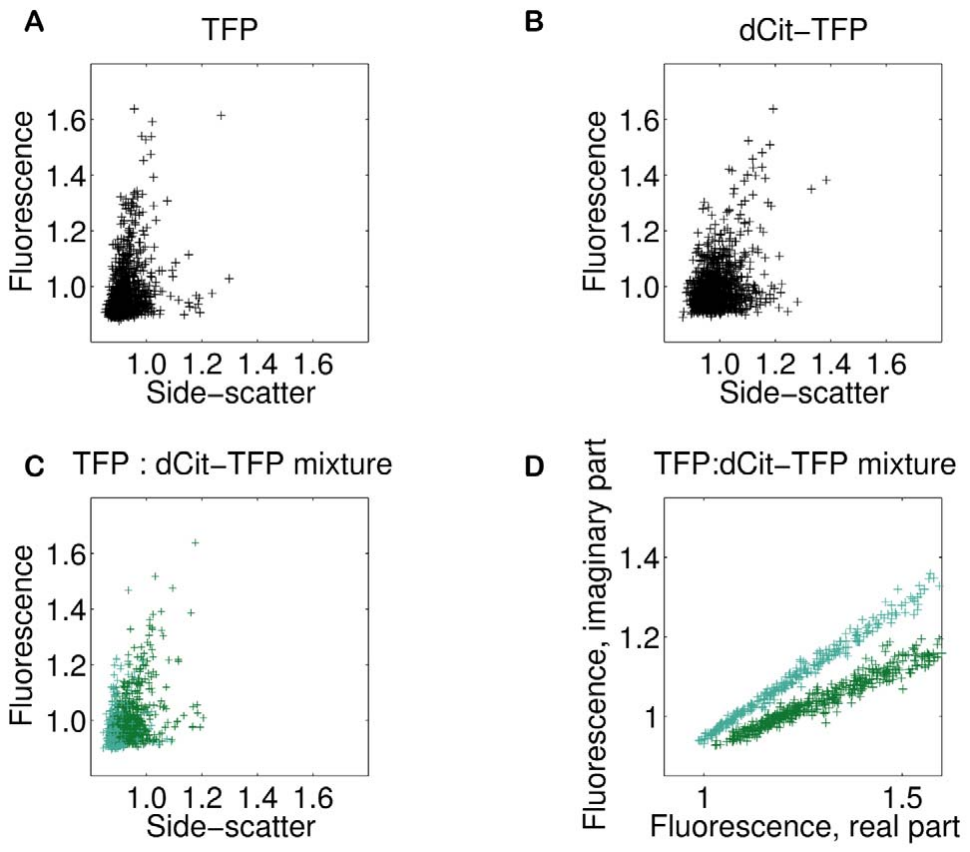
Detecting lifetime differences in flow. A) Intensity scatter plot for the BSY034 TFP strain. B) Intensity scatter plot for the BSY035, the dCit-TFP strain. C) Intensity scatter plot for mixture of BSY034 and BSY035, colored according to phase. Green dots represent phase delays of less than 30 degrees, blue dots, phase delays more than 30 degrees. D) Pseudophasor plot of the data from 3C. Supplemental Figure S4 shows the same plot but with a red/green color scheme.

Figure 3A shows a dot plot of the intensity of the fluorescence emission vs. side-scattered light from a population of cells expressing TFP. Figure 3B shows a similar plot for cells expressing dCit-TFP. Figure 3C shows a similar plot for a mixture of cells expressing TFP and cells expressing dCit-TFP, with the dots colored to indicate the phase delay: (light blue) has less than 30 degrees and (dark green) has more than 30 degrees. The light blue and dark green dots are intermingled. Figure 3D shows a pseudophasor plot of the same data. The dots representing the cell sub-populations are well-separated. A histogram generated from the data in Figure 3D shows 2 distinct peaks corresponding to 2 separate fluorescence lifetimes in the mixture (Figure S3).

**Figure 4:**
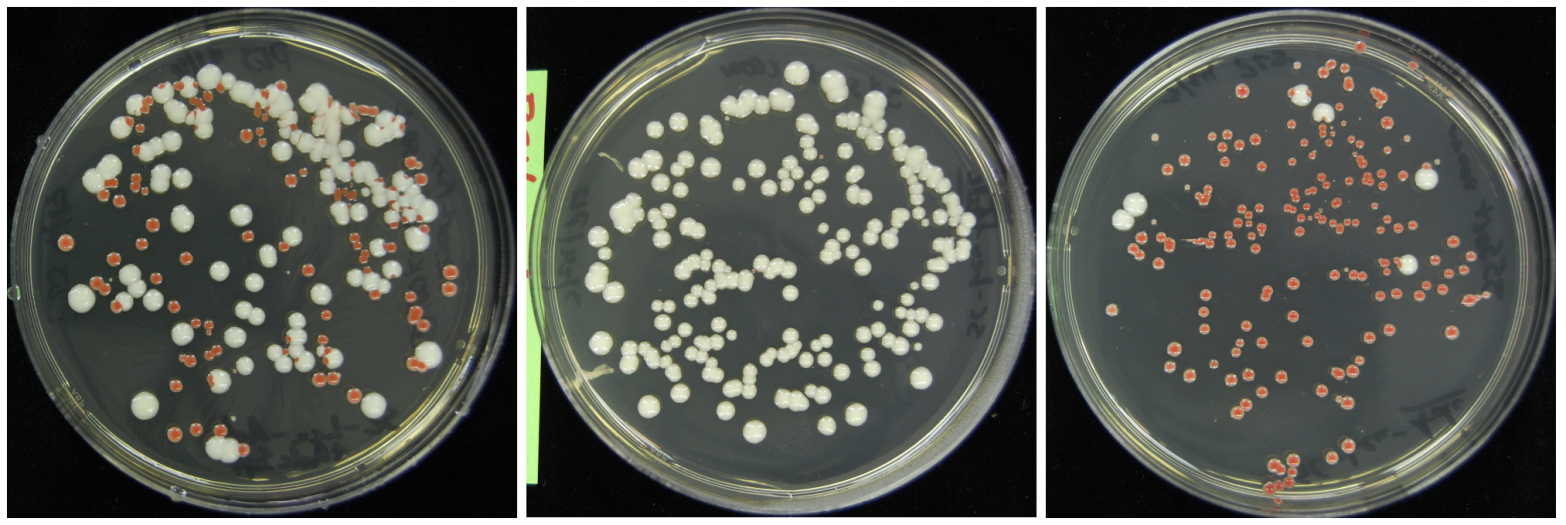
Sorting yeast cells by fluorescence lifetime. We plated cells from pre-sort mixture and sorts on SC-leu- with 0.001% adenine, and photographed colonies after 48 hours. Left panel, pre sort mixture. Middle panel: sorted for long lifetime, enriched for white (BSY034, TFP) colonies. Right panel, sorted for shortened lifetime, enriched for red (BSY035, dCit-TFP) colonies. For each of three different trials we sorted three or four plates each of TFP and dCit-TFP strains. The plates here are representative examples.

We used this phase difference to sort the mixture into nearly pure subpopulations with different lifetimes (Figure 4). We drew gates around areas of pseudophasor plots, like those in supplemental figure S2, that corresponded to pure populations of cells, and ran through the sorter a 1∶1 mixture of these cells. We plated onto low adenine medium cells from the 1∶1 mixture before each sort in order to confirm the starting ratio (Figure 4 left panel) and after each sort (Figure 4 middle and right). We incubated plates until we could count visible colonies. We defined sorting purity as the percentage of the cells deflected into the sort bin that are of the correct strain. In three experiments, with at least three runs for each strain, we achieved average sorting purities of 97.1% for the TFP strain and 93.7% for the dCit-TFP strain. Table 3 lists the data from all experiments.

**Table 3:** Sorting Data.

Trial^1^	Sorted Strain	Colonies^2^	Purity^3^
**1**	BSY034	146	98.6±0.57
	BSY035	98	96.0±1.68
**2**	BSY034	188	97.3±0.63
	BSY035	126	94.6±3.8
**3**	BSY034	97	95.4±3.2
	BSY035	66	90.4±1.5
**Aggregate**	BSY034	144	97.1±1.62
	BSY035	97	93.7±2.9

1 Each trial was composed of 3 or 4 runs.

2 average number of individual colonies per plate.

3 Percentage of correctly sorted cells ± standard deviation.

## Discussion

Over the past ten years, a substantial body of work has demonstrated the utility of flow cytometric measurements of cell signaling. These permit quantification of signaling proteins from fixed cells probed with fluorescent antibodies. Such studies have allowed identification of cell subpopulations that signal differently [1], [60]. Considerable attention to new methods of analysis has allowed researchers to use such data to make valid inferences about cause and effect relationships among measured variables [38], to canvass key changes in signaling caused by treatment with inhibitors of specific signaling proteins [61] and to infer order of events and developmental trajectories for different cell types from collections of static measurements or snapshots [62].

Here, we demonstrated the ability of flow cytometry to determine fluorescence lifetimes of XFPs in live cells, and to isolate cell subpopulations by sorting on lifetime. We imagine a number of ways that this new capability might positively impact future studies of cell signaling.

The first is due to the fact that light signals from XFPs are weak. Because the lifetime of a fluorophore depends on its immediate environment, changes in lifetime, for example gain in donor fluorophore lifetime after dissociation of a FRET pair, can sometimes supplement information from change in the intensity of fluorescence signal. The ability to believe weaker signals should facilitate studies of signaling events using fluorescent proteins present in physiologically appropriate abundance.

The second is due to the fact that the emission spectra of different XFPs are broad and overlapping. Some work, including formation of hypotheses about order of events, developmental trajectories, and relationships of cause and effect mentioned above, benefits from the ability to distinguish>12 different signals, provided by antibodies tagged either with chemical fluorophores [1], [60], or isotopic mass tag barcodes [61], from each cell. Yet the broad emission spectra of different XFPs operationally limits the number of distinguishable color signals from living cells to 5 or 6. Ability to distinguish signals from XFPs with similar emission but different lifetimes increases the number of distinguishable signals from living cells. The ability to study sorted subpopulations of these cells after measurement should enable further experimentation to test these hypotheses.

The third consequence of this research is due to the fact that the capabilities described here will allow isolation of subpopulations of cells with signaling reporters whose XFP fluorophores have different lifetimes indicating different signaling states. Work by us and others has demonstrated that single cells exist in different physiological states defined by significant, weakly heritable differences in their ability to send and respond to signals [63]. Recall that the molecular events involved in signaling can change the atomic environment of XFP fluorophores in reporter cells, for example by causing dissociation of a FRET pair, as above, or by translocation to a different, perhaps more crowded, subcellular location, in which fluorophore lifetime is shortened [64]. In these instances, ability to sort cell subpopulations by differences in lifetime might allow isolation of subpopulations of live cells in different signaling states and their subsequent analysis. For example, a greater ability to sort cells based on signaling differences might allow identification of subpopulations of tumor or immune system cells that signal differently, and subsequent molecular and functional study to understand the causes of their different physiological states.

## Materials and Methods

### Nucleic acid manipulation and plasmid construction

We generated plasmids based on the yeast shuttle vector pADNS which contains the 2u high copy yeast origin of replication, the Leu2 gene for selection on leucine deficient minimal media and the yeast ADH1 promoter and terminator sequences [56]. For PCR reactions, we used Phusion polymerase (NEB) and followed the recommended reaction and cycling conditions. For site-directed mutagenesis we used the Quick-change Multisite kit following the manufacturer's directions (Stratagene). All primers are listed in Table S1. We amplified the gene for yECitrine from plasmid pKT140 [65] using primers BS1 and BS2, which added 5′ HindIII and 3′ NotI sites, and cloned the PCR fragment into pCR2.1Topo (Invitrogen). Next, we constructed 2 additional Citrine variants by site-directed mutagenesis with the following primers: BS3 and BS4 (F46L, A206K) to generate ymECitrine_F46L_ and primers BS3 BS5 BS6 (F46L, Y145W, H148V, F223R) to generate dark Citrine.

We cloned the genes for ymECitrine_F46L_ and dark Citrine into plasmid pADNS from HindIII to Not1 which generated plasmids pBS4(ymECitrineF46L) and pBS19(dark Citrine). We amplified yeast codon optimized, monomeric teal fluorescent protein [53] from a synthetic DNA block(DNA2.0) with primers BS7 and BS8, digested the PCR fragment with HindIII and Not1 and cloned the digested fragment into pADNS resulting in plasmid pBS6.

To generate the fusion XFPs, we amplified darkCitrine from pBS19 with primers BS9 and BS10 which added 5′ and 3′ HindIII sites and a 3′GSGG in-frame linker, digested the PCR product with HindIII, and cloned it into the HindIII site of plasmid pBS3 or pBS4 to generate pBS24(dCitrine-mTFP1) and pBS32(dCitrine-ymECitrine_F46L_) respectively.

### Yeast strain construction and culture

We list strains used in the study in Table 1. We grew parental strains BY4741, the ADE+ strain WY581 and the ADE- strain WY639 by standard methods in YPAD. WY639, a gift from Dr. Wenying Shou, contains a deletion of the entire *ADE2* coding sequence which is replaced with a *HISG+* marker, and thus does not revert. We grew plasmid transformed strains in synthetic complete media with glucose and lacking leucine (SC-Leu). For colony counting assays, we supplemented SC-Leu with 0.001% adenine. We transformed yeast strains with plasmid DNA by standard methods [52].

For microscopy, we streaked yeast strains from -80o frozen stocks onto SC-LEU plates and incubated at 30° for 48 hours. We picked single yeast colonies off each plate with a sterile pipet tip. We then lightly touched the tip to deposit cells onto well bottoms in uncoated 96-well, glass bottom plates pre-filled with 100 ul of SC-LEU. We incubated the plates at 30° for at least 2 hours. During this time the cells continued to divide. We used an epifluorescence microscope to verify that cells were fluorescent before FLIM microscopy.

### Measurement of emission spectra from engineered proteins

We grew strains expressing XFPs from starter cultures from single yeast colonies in 5 ml of SC-Leu overnight at 30 d with shaking at 250 rpm. The next day we split cultures 1 to 10,000 into 1 well of a glass bottom 96 well plate in 100 ul of fresh SC-Leu and grew for two hours at 30 C. We conducted spectral scans on a Zeiss LSM780 confocal microscope with a 40× objective and wide open pinhole and excited the samples using the 458 nm line from an Argon laser. We captured the emission spectra from the cells over a wavelength range of 460–650 nm in 8.9 nm bins (peak excitation/emission for mTFP-1 is 462/492nm and 516/528nm for Citrine). We processed the emission spectra in Matlab by normalizing to an estimate of the peak intensity, such that the peak value displayed for each spectrum was always unity as follows. We estimated the peak intensity, and the wavelength at which it occurred, by interpolating a quadratic function through the three neighboring wavelength bins which had the highest recorded spectral intensities. We included this estimated peak intensity point in the data plotted in Figure 1B.

### Microscopic measurements of fluorescence lifetime

We carried out lifetime measurements, on cells prepared as described in the previous section, on two FLIM systems. Both systems utilized a Nikon Eclipse inverted microscope. We call the first of these, an array scanning confocal system, custom-built by Visitech Ltd, FLIM-c. We call the other, a widefield system built by Lambert Instruments, FLIM-w. For both systems, we modulated both the illumination and the camera gain at 30 MHz.

FLIM-c used a 442 nm laser (to excite TFP and dCit-TFP), and a 514 nm laser (to excite Citrine and dCit-Cit). FLIM-c employed an ICCD camera (Lambert LI2-CAM). We used a 60X 1.4 NA oil immersion objective, an ICCD gain setting of 650–750 volts, depending on sample brightness, and captured images using an appropriate triple-bandpass emission filter (Chroma 69008 m).

FLIM-w used 468 nm LEDs to excite TFP and dCit-TFP strains. We used a 40X NA 0.75 air objective, an ICCD gain setting of 650 Volts, a cathode bias of −0.2 V, and appropriate exposure times as needed, always less than 1 s. We used Lambert Instruments' proprietary LIFA software to capture and process all images.

Prior to recording images on either system, we chose visual fields that contained only a few, well-separated cells.

To generate the measurements in Table 2 and the lifetime images in Figure 2, we measured the lifetime of each sufficiently bright pixel within the field-of-view. We accomplished this by capturing sets of images in rapid succession, with each set consisting of multiple images of the same field of view. We captured each member of these sets using a different relative phasing of the modulated camera gain with respect to the modulated illumination; we called such sets of images “phase stacks”. We also recorded phase stacks from reference solutions with known fluorescence lifetimes (100 *µ*M Erythrosin-B, 0.086 ns, Sigma-Aldrich 198269-25G, and 10 *µ*M Fluorescein, 4.0 ns, Lambert Instruments).

On FLIM-w, the LIFA software performed the task of computing lifetime images from phase stacks of pixels corresponding to cells, and it reported the average values which we display in Table 2.

On FLIM-c, we extracted the raw image data and processed it ourselves using open source software we developed in Matlab. We exported the phase stacks from the proprietary VoxCell software (Visitech Ltd.) as Tagged Image File Format (TIFF) files, in 10-page 16-bit uncompressed grayscale format. Each page comprised the FOV in 520×696 pixels, and corresponded to one particular value of the phase difference between the ICCD camera gain and the illumination intensity. There were 10 such phase difference values, spaced by 36 degrees. We refer to the 10 intensity values corresponding to a particular location in the FOV as a stack column. We used each stack column that contained sufficient counts to generate a measurement of illumination-to-emission phase difference at each sufficiently bright pixel in the FOV. We determined the number of counts that was sufficient on a case-by-case basis, by comparing the typical brightness of pixels within cells to that of background pixels and selecting a value that excluded non-cell pixels. This step would not be difficult to automate, but we did not do so.

We took Fast Fourier Transforms (FFTs) as defined in [66], along the 10 points of phase dimension of the cell and reference stacks, and thus generated five informative complex coefficients for each stack column in both the reference and cell stacks. We used the second FFT coefficient, the one associated with the fundamental modulation frequency of the excitation (here, 30 MHz) to compute the phase offsets 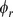 and 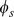 for the reference and cell stacks respectively. The formula for the lifetime 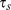 of a sample having a simple decay is then
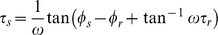
where 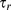 is the lifetime of the reference. To make fluorescence lifetime images, we computed 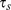 for each column in a phase stack, and displayed these numbers as colors.

### Phasor analysis for instrument diagnosis

We used phasor analysis to troubleshoot FLIM-c and the flow cytometer. A phasor is a single complex number that captures both the loss of modulation depth (demodulation) and the phase delay of fluorescent emission resulting from sinusoidally modulated excitation (See [51] for an introduction to these methods). To measure phasors on FLIM-c, we took the FFT coefficients, computed as described in the previous section, and normalized them by dividing by the first FFT coefficient, which is essentially the average emission intensity recorded in a phase stack. We then used the reference stack to determine a complex correction factor for phase delays within the instrument, such that the swarm of points plotted in the complex plane (corresponding to the phasors corresponding to the modulated fluorescence of the reference fluorophore) fell properly into place near the correct position on the universal circle (see below). To measure phasors from the BD FACSVantage, we collected and focused the excitatory light (by side scatter) and the emitted fluorescence onto photomultiplier tubes (PMTs), thus converting these light signals to analog currents. We used an Innovative Integration X5-210 M FPGA-based data collection system (here called BlackBart), to digitize and record these currents. We conformed to an important requirement for the digitization: the sampling rate must be an integral multiple of the modulation frequency (here, 250 MHz and 25 MHz, respectively); for a periodic signal, this requirement compresses the signal information, expressed as an FFT, into the smallest possible number of FFT coefficients (those corresponding to integral multiples of the modulation frequency). We computed phasors offline from these digitally recorded signals, using additional Matlab software. For each cell, we did so by first computing FFTs of appropriate subsets of both signals (such a subset consists of an integral number of modulation cycles with detected signal). Since we digitized ten points per modulation cycle, the modulation frequency corresponded to the tenth non-zero-frequency component of the FFT, i.e. to the eleventh FFT coefficient. Thus, we took the ratio of the eleventh FFT coefficients from the emission divided by the eleventh FFT coefficient of the excitation, each normalized by its first FFT coefficient (corresponding to zero frequency).

Phasors computed in this way, using data recorded from a fluorophore with a simple monoexponential decay, when plotted in the complex plane, will necessarily lie (within instrumental error) on a semi-circle centered at ½ + 0**i**, with a radius of ½. This is referred to as the “universal circle” [51]. We thus plotted phasors from many cells as points in the complex plane and used, when we observed it, any systematic deviation from the universal circle to generate a complex correction factor, such that the swarm of plotted points landed on the universal circle.

### Pseudophasor analysis for cell sorting

To sort cells according to the fluorescence lifetimes of their XFPs, we developed a method which we named “pseudophasor analysis.” This method comprises the computation of complex numbers called pseudophasors, their plotting in the complex plane, and the drawing of a polygon to select a desired subset of them during sorting. We computed a pseudophasor immediately after the detection of each fluorescent cell event.

A pseudophasor is similar to a phasor, in that its argument is the phase delay, but its magnitude is proportional to the peak to trough amplitude of the intensity fluorescence emission (rather than equal to the demodulation). Plots of pseudophasors reveal useful information about the phase delay and amplitude of the modulation of the XFP fluorescence. The phase delay is a measure of the fluorescence lifetime, while the amplitude can reveal instrumental issues and provides an additional sorting criterion.

Our pseudophasors are equivalent to the complex FFT coefficient corresponding to the modulation frequency. However, we did not compute them using an FFT, but instead by using the Goertzel algorithm [67]. This algorithm is a “recursive digital filter”, i.e. it operates on digitized time series data, using a weighted sum of previous outputs as well as previous inputs to compute the current output. (See Figure S5 for a representative implementation of the Goertzel algorithm in Matlab). Unlike the FFT, the Goertzel algorithm can operate on each new data point as it is captured, and it returns a result corresponding to a single user-selected frequency- in our case, the modulation frequency. Thus, the Goertzel algorithm requires minimal memory and computes no extraneous values. In the aforementioned weighted sum, the values of the weights select the frequency for which the algorithm yields the corresponding pseudophasors. The reciprocal of the user-selected frequency is an integral multiple of the sampling period (here (250 MHz)^−1^, or 4 ns). This algorithm can be computed in less time than an FFT.

We hard-coded the Goertzel algorithm on the Innovative Integration X5-201M FPGA for a modulation frequency of 25 MHz, i.e. 10 sampling periods, to give the pseudophasors directly. We plotted and show examples of the real and imaginary parts of these pseudophasors in figure 3D. Our implementation allowed isolation of more than 140 cells from minority subpopulations per second.

### Flow Cytometry and sorting

We streaked frozen yeast cultures onto SC-leu agar plates and incubated at 33°C for 48 hr. We inoculated cells from a single colony into 1.5 ml of SC-Leu liquid and incubated at 30°C for 12 hours with shaking at 300 rpm. The next day we inoculated 200 ul of each overnight culture into 1.8 ml of fresh SC-Leu liquid and grew for 3 hours at 30°C with shaking at 300 rpm. We harvested cultures by centrifugation for 5 min at 1100 rpm in a table top centrifuge and resuspended the cell pellet in 3 ml of SC-leu. We then ran 200 ul aliquots on an Accuri cytometer to determine concentration and diluted the final samples to 2 million cell/ml in SC-Leu.

We ran samples on a modified BD FACSVantage SE calibrated for optimal signal on the SSC channel (Semrock 448/20 filter) and the FL1 channel (Semrock 458LP filter) with Beckman Coulter Flow-Check microspheres. We illuminated them using a Coherent OBIS 445 nm diode laser modulated with a 25 MHz sinusoid generated by Tektronix AFG-3102 function generator. We rerouted the PMT outputs from the SSC and FL1 channels through DC-100 high speed pre-amplifiers (ARI Corp.) to BlackBart, an Innovative Integration X5-210M FPGA-based data collection system running KYTOS analysis software (DarklingX, Los Alamos, NM). We optimized the incoming signals using a virtual oscilloscope in KYTOS. We nulled the instrumental phase delay for each experiment using Beckman Coulter Flow-Check beads [47].

Following an event trigger based on side-scattered light, BlackBart begins computing a pseudophasor for the event using captured waveform data. If the pseudophasor falls within the sort gates, BlackBart generates a sort pulse and sends it to the FACSVantage on an otherwise unused fluorescence channel (FL2). Many cells expressing dCit-TFP, and some expressing TFP, were too dim to trigger BlackBart; such cells were neither detected nor sorted.

In normal sorting, the FACSVantage generates a sorting decision that is effectively coincident in time with the event trigger, and relays that decision to the downstream sorting apparatus. The sorting apparatus essentially counts droplets, and activates the deflector to select the right one. Here, we ran the FACSVantage at a droplet generation frequency of 17.7 kHz, and thus generated a new droplet every 57 microseconds; we refer to the latter as the “droplet delay time”. In normal operation (as opposed to lifetime sorting), we activated the deflector at delay times corresponding to between 8 and 13 droplets, following an event trigger that met the easily-computed sorting criteria. We set the droplet delay using fluorescent microspheres, prior to running cells.

For lifetime sorting, we needed to account for the time to compute pseudophasors and make the sorting decision. This time depends on a number of unpredictable instrumental factors, but was almost always substantially less than 50 microseconds. Thus, advancing the deflection timing by one additional droplet generation time sufficed to compensate for the time spent in sorting.

To sort, we first ran dCit-TFP yeast to set the PMT gain for optimal yeast detection. We then ran pure populations of dCit-TFP and TFP and generated pseudophasor plots as described above. We used these plots to draw polygons in the complex plane, one for each strain, to use as non-overlapping gates for sorting. We then sorted mixtures of BSY034 (TFP) and BSY035 (dCit-TFP), using those gates. Cells were sorted directly onto pre-warmed SC-Leu plates containing 0.001% adenine. We counted colonies after 48 hours.

## Supporting Information

Figure S1 **Diagram of the modified cytometer.** Simplified diagram of the flow cytometric lifetime detection/sorting system. Yeast expressing fluorescent proteins rapidly transit a modulated laser beam from a diode laser, driven by a sine wave from a function generator at RF frequencies, following hydrodynamic focusing within the cytometer nozzle. Scattered laser light and fluorescence are collected 90° incident to the laser excitation beam and are optically focused, split, and filtered onto photomultiplier tube (PMT) detectors. The resultant photocurrent signal is amplified utilizing high frequency PMT preamplifiers that do not attenuate the modulated Gaussian shaped signal. These signals are sampled by the BlackBart data acquisition/analysis system, which relays cytometric information (pulse height, width) as well as pulse waveforms to a host computer running KYTOS analysis software. From KYTOS the phase shift between the two signals, the fluorescence lifetime, and pseudophasor information can be analyzed and recorded. Sort gates can also be set within the pseudophasor plot which are relayed back to the BlackBart system, where an FPGA based analysis analogous to that happening on KYTOS will be performed. When events occur with pseudophasor parameters that fall within the predefined sort gates, sorting pulses are relayed to the FACSVantage sorting system. The FACSVantage drives the drop drive control in which a piezoelectric device oscillates at a specific frequency and imparts vibration into the fluid stream that causes droplets to form. When an event that falls within the sorting gates is detected the FACSVantage sorting system will charge the fluidic stream right before the moment of droplet breakoff, leaving the resultant drop with an electrical charge. As the charged droplet containing the particle of interest falls, it passes through two strongly charged deflection plates. The charged particle will be attracted/deflected one way depending on its charge. These cells of interest are collected in a bin separate from the unwanted cells, which are uncharged and fall down to a central waste containment aspirator.
(PDF)

Figure S2 **Pseudophasor sort gates.** Screen capture of a pseudophasor plot produced on the FACSVantage, with operator-drawn polygonal sort gates.
(TIF)

Figure S3 **Histograms from mixed cell populations.** Data from 1000 events, for a mixture of cells expressing TFP and cells expressing dCit-TFP. A) Phase delay of fluorescence signal in degrees, showing two clear peaks. B) Fluorescence intensity in arbitrary units, showing only one peak.
(TIF)

Figure S4 **Detecting lifetime differences in flow.** A) Intensity scatter plot for the BSY034 TFP strain. B) Intensity scatter plot for the BSY035, the dCit-TFP strain. C) Intensity scatter plot for mixture of BSY034 and BSY035, colored according to phase. Green dots represent phase delays of less than 30 degrees, red dots, phase delays more than 30 degrees. D) Pseudophasor plot of the data from 3C.
(TIF)

Figure S5 **Goertzel algorithm.** Matlab code implementing the Goertzel algorithm.
(M) Click here for additional data file.

Figure S6 Feb12.zip: Flow cytometry data archive, including waveform data (*.csv) as well as list mode data (*.fcs), for pure BSY034, pure BSY035, and a mixture of the two.
(ZIP)

Figure S7 Apr01_34.zip: Flow cytometry data archive, including waveform data (*.csv), list mode data (*.fcs), for pure BSY034.
(ZIP)

Figure S8 Apr01_35.zip: Flow cytometry data archive, including waveform data (*.csv) as well as list mode data (*.fcs), for pure BSY035.
(ZIP)

Figure S9 Apr01_Mix.zip: Flow cytometry data archive, including waveform data (*.csv) as well as list mode data (*.fcs), for a mixture of BSY034 and BSY035.
(ZIP)

Figure S10 Apr29.zip: Flow cytometry data archive, including waveform data (*.csv) as well as list mode data (*.fcs), for pure BSY034, pure BSY035, and a mixture of the two.
(ZIP)

Figure S11 **TFP.zip: FLIM data archive with TIFF phase stacks of BSY034 cells, reference phase stacks of Erythrosin-B solution, and data format documentation.**
(ZIP)

Figure S12 **dCitTFP.zip: FLIM data archive with TIFF phase stacks of BSY035 cells, reference phase stacks of Erythrosin-B solution, and data format documentation.**
(ZIP)

Figure S13 **Cit.zip: FLIM data archive with TIFF phase stacks of BSY004 cells, reference phase stacks of Erythrosin-B solution, and data format documentation.**
(ZIP)

Figure S14 **dCitCit.zip: FLIM data archive with TIFF phase stacks of BSY015 cells, reference phase stacks of Erythrosin-B solution, and data format documentation.**
(ZIP)

Table S1 **Primer Sequences.** Primers used to engineer XFP constructs.
(PDF)

## References

[1] Irish JM, Hovland R, Krutzik PO, Perez OD, Bruserud Ø, et al (2004) Single Cell Profiling of Potentiated Phospho-Protein Networks in Cancer Cells. Cell 118: 217–228. 15260991 10.1016/j.cell.2004.06.028

[2] Irish JM, Myklebust JH, Alizadeh AA, Houot R, Sharman JP (2010) B-cell signaling networks reveal a negative prognostic human lymphoma cell subset that emerges during tumor progression. Proc Natl Acad Sci U S A 107: 12747–12754. 20543139 10.1073/pnas.1002057107 PMC2919949

[3] Palazzo AL, Evensen E, Huang YH, Cesano A, Nolan G, et al (2011) Association of reactive oxygen species-mediated signal transduction with in vitro apoptosis sensitivity in chronic lymphocytic leukemia B cells. PLos ONE 6: 1–13. 10.1371/journal.pone.0024592 PMC3189964 22016760

[4] Du J, Wang J, Kong G, Jiang J, Zhang J, et al (2012) Signaling profiling at the single cell level identifies a distinct signaling signature in murine hematopoietic stem cells. Stem Cells 30: 1447–1454. 22628264 10.1002/stem.1127 PMC3438669

[5] Schnell U, Dijk F, Sjollema KA, Giepmans BNG (2012) Immunolabeling artifacts and the need for live-cell imaging. Nature Methods 9: 152–158. 22290187 10.1038/nmeth.1855

[6] Brent R (2009) Cell signaling: What is the signal and what information does it carry? FEBS Lett 583: 4019–4024. 19917282 10.1016/j.febslet.2009.11.029

[7] Albeck JG, Mills GB, Brugge JS (2013) Frequency-modulated pulses of ERK activity transmit quantitative proliferation signals. Molecular Cell 49: 249–261. 23219535 10.1016/j.molcel.2012.11.002 PMC4151532

[8] Chudakov DM, Matz MKV, Lukyanov S, Lukyanoc KA (2010) Fluorescent Proteins and Their Applications in Imaging Living Cells and Tissues. Physiol Rev 90: 1103–1163. 20664080 10.1152/physrev.00038.2009

[9] Yu R, Gordon A, Colman-Lerner A, Benjamin KR, Pincus D, et al (2008) Negative feedback optimizes information transmission in a cell signaling system. Nature 456: 755–761. 19079053 10.1038/nature07513 PMC2716709

[10] Bush A, Colman-Lerner A (2013) Quantitative measurement of protein relocalization in live cells. Biophys. J 104: 727–736. 23442923 10.1016/j.bpj.2012.12.030 PMC3566451

[11] Horgan AM, Stork PJ (2003) Examining the mechanism of Erk nuclear translocation using green fluorescent protein. Exp Cell Res 285: 208–220. 12706116 10.1016/s0014-4827(03)00037-5

[12] Plotnikov A, Zehorai E, Procaccia S, Seger R (2011) The MAPK cascades: Signaling components, nuclear roles and mechanisms of nuclear translocation. Biochimica Biophysica Acta 1813: 1619–1633. 10.1016/j.bbamcr.2010.12.012 21167873

[13] Yi TM, Kitano H, Simon MI (2003) A quantitative characterization of the yeast heterotrimeric G protein cycle. Proc Natl Acad Sci USA 100: 10764–10769. 12960402 10.1073/pnas.1834247100 PMC196877

[14] Gadella TWJ (2009) FRET and FLIM techniques, Laboratory Techniques in Biochemistry and Molecular Biology. Volume 33 . Amsterdam: Elsevier Press.

[15] Kasai RS, Kasumi A (2014) Single-molecule imaging revealed dynamic GPCR dimerization. Curr Opin Cell Biol 27: 78–86. 24480089 10.1016/j.ceb.2013.11.008

[16] Sample V, Mehta S, Zhang J (2014) Genetically encoded molecular probes to visualize and perturb signaling dynamics in living biological systems. J Cell Science 127: 1151–1160. 24634506 10.1242/jcs.099994 PMC3953811

[17] Mochizuki N, Yamashita S, Kurokawa K, Ohba Y, Nagai T, et al (2001) Spatio-temporal images of growth-factor induced activation of Ras and Rap1. Nature 411: 1065–1068. 11429608 10.1038/35082594

[18] Nakamura T, Aoki K, Matsuda M (2005) Monitoring spatiotemporal regulation of Ras and Rho GTPases with GFP-based FRET probes. Methods 37: 146–153. 16288890 10.1016/j.ymeth.2005.05.021

[19] Kiyokawa E, Aoki K, Nakamura T, Matsuda M (2011) Spatiotemporal regulation of small GTPases as revealed by probes based on the principle of forster resonance energy transfer (FRET): implications for signaling and pharmacology. Ann. Rev. Pharm. Tox 51: 337–358. 10.1146/annurev-pharmtox-010510-100234 20936947

[20] Zhang J, Ma Y, Taylor SS, Tsien RY (2001) Genetically encoded reporters of protein kinase A activity reveal impact of substrate tethering. Proc Natl Acad Sci USA 98: 14997–15002. 11752448 10.1073/pnas.211566798 PMC64972

[21] Ting AY, Kain KH, Klemke RL, Tsien RY (2001) Genetically encoded fluorescent reporters of protein tyrosine kinase activities in living cells. Proc Natl Acad Sci USA 98: 15003–15008. 11752449 10.1073/pnas.211564598 PMC64973

[22] Violin JD, Zhang J, Tsien RY, Newton AC (2003) A genetically encoded fluorescent reporter reveals oscillatory phosphorylation by protein kinase C. J. Cell Bio 161: 899–909. 12782683 10.1083/jcb.200302125 PMC2172956

[23] Fuller B, Lampson M, Foley EA, Rosasco-Nitcher S, Le KV, et al (2008) Midzone activation of aurora B in anaphase produces an intracellular phosphorylation gradient. Nature 453: 1132–1136. 18463638 10.1038/nature06923 PMC2724008

[24] Harvey CD, Ehrhardt AG, Cellurale C, Zhong H, Yasuda R, et al (2008) A genetically encoded fluorescent sensor of ERK activity. PNAS 105: 19264–19269. 19033456 10.1073/pnas.0804598105 PMC2614750

[25] Zhou X, Herbst-Robinson KJ, Zhang J (2012) Visualizing Dynamic Activities of Signaling Enzymes Using Genetically Encodable Fret-Based Biosensors: From Designs to Applications in Imagine and spectrocopic analysis of living cells— optical and spectroscopic techniques. Methods in Enzymol 504 p317–340. 10.1016/B978-0-12-391857-4.00016-1 PMC4384881 22264542

[26] Ekimov AI, Onushchenko AA (1981) Quantum size effect in three-dimensional microscopic semiconductor crystals. JETP Lett 34: 345–349.

[27] Resch-Genger U, Grabolle M, Cavaliere-Jaricot S, Nitschke R, Nann T (2008) Quantum dots versus organic dyes as fluorescent labels. Nature Methods 5: 763–775. 18756197 10.1038/nmeth.1248

[28] Chattopadhyay PK, Perfetto SP, Yu J, Roederer M (2010) The use of quantum dot nanocrystals in multicolor flow cytometry. In *Wiley Interdiscip Rev Nanomed Nanobiotechnol* 2 (4), pp.334–348. DOI:10.1002/wnan.75. 10.1002/wnan.75 20101649

[29] Akinfleva O, Nabiev I, Sukhanova S (2013) New directions in quantum dot-based ctyometry detection of cancer serum markers and tumor cells. Crit Rev Oncol Hematol 86: 1–14. 23058250 10.1016/j.critrevonc.2012.09.004

[30] Shaner NC, Steinbach PA, Tsien RY (2005) A guide to choosing fluorescent proteins. Nature Methods 2: 905–909. 16299475 10.1038/nmeth819

[31] Thomson TM, Benjamin KR, Bush A, Love T, Pincus D, et al (2011) Scaffold number in yeast signaling system sets tradeoff between system output and dynamic range. Proc Natl Acad Sci USA 108: 20265–20270. 22114196 10.1073/pnas.1004042108 PMC3250143

[32] Monici M (2005) Cell and tissue autofluorescence research and diagnostic applications. Biotechnol Annu Rev 11: 227–256. 16216779 10.1016/S1387-2656(05)11007-2

[33] Gordon A, Colman-Lerner A, Chin TE, Benjamin KR, Brent R (2007) Single-cell quantification of molecules and rates using open source microscope based cytometry. Nature Methods 4: 175–181. 17237792 10.1038/nmeth1008

[34] Houston JP, Sanders CK, Trujillo A, Naivar MA, Freyer JP (2009) Measurement of modulated autofluorescence signals in flow cytometry. IFMBE Proceedings 24: 261–262.

[35] Shapiro HM (2003) Practical flow cytometry. Hoboken, New Jersey: John Wiley and Sons.

[36] Lackowicz JR (2006) Principles of fluorescence spectroscopy. 3rd edition. New York, New York: Springer.

[37] Pearl J (2000) Causality: models, reasoning, and inference. Cambridge, UK: Cambridge University Press.

[38] Sachs K, Perez O, Pe'er D, Lauffenburger DA, Nolan GP (2005) Causal protein-signaling networks derived from multiparameter single-cell data. Science 308: 523–529. 15845847 10.1126/science.1105809

[39] Brent R, Lok L (2005) A fishing buddy for hypothesis generators. Science 308: 504–506. 15845840 10.1126/science.1110535

[40] Sachs K, Gentles AJ, Youland R, Itani S, Irish J, et al (2009) Characterization of patient specific signaling via augmentation of Bayesian networks with disease and patient state nodes. Conf Proc IEEE Eng Med Biol Soc 2009: 6624–6627. 19963681 10.1109/IEMBS.2009.5332563 PMC3124088

[41] Spencer RD, Weber G (1969) Measurements of subnanosecond fluorescence lifetimes with a cross-correlation phase fluorometer. In *Ann NY Acad Sci* 158: 361–376.

[42] van der Pol B (1946) The fundamental principles of frequency modulation. In Journal of the Institution of Electrical Engineers - Part III: Radio and Communication Engineering 93: 153–158.

[43] Pinsky BG, Ladasky JJ, Lakowicz JR, Berndt K, Hoffman RA (1993) Phase-resolved fluorescence lifetime measurements for flow cytometry. Cytometry 1: 123–135. 10.1002/cyto.990140204 8440147

[44] Steinkamp JA, Yoshida TM, Martin JC (1993) Flow cytometer for resolving signals from heterogeneous fluorescence emissions and quantifying lifetime in fluorochrome-labeled cells/particles by phase-sensitive detection. Review of Scientific Instruments 64: 3440–3450.

[45] Steinkamp JA, Chrissman HA (1993) Resolution of fluorescence signals from cells labeled with fluorochromes having different lifetimes by phase-sensitive flow cytometry. Cytometry 14: 210–216. 8440154 10.1002/cyto.990140214

[46] Steinkamp JA, Parson JD (2001) Flow cytometric, time-resolved measurements by frequency heterodyning of fluorescence emission signals. Proc Intern Soc Optical Eng 4260: 166–174.

[47] Cao R, Pankayatselvan V, Houston JP (2013) Cytometric sorting based on the fluorescence lifetime of spectrally overlapping signals. Optics Express 21: 14816–14831. 23787669 10.1364/OE.21.014816 PMC3726248

[48] Houston JP, Naivar M, Freyer JP (2010) Digital Analysis and Sorting of Fluorescence Lifetime by Flow Cytometry. Cytometry A, Volume 77A: 861–872. 10.1002/cyto.a.20930 PMC2930036 20662090

[49] Naivar MA, Parson JD, Wilder ME, Habbersett RC, Edwards BS, et al (2007) Open, reconfigurable cytometric acquisition system: ORCAS. Cytometry A 71: 915–924. 17680705 10.1002/cyto.a.20445

[50] Naivar MA, Wilder ME, Habbersett RC, Woods TA, Sebba DS, et al (2009) Development of small and inexpensive digital data acquisition systems using a microcontroller-based approach. Cytometry A 75: 979–989. 19852060 10.1002/cyto.a.20814 PMC3969846

[51] Digman MA, Caiolfa VR, Zamai M, Gratton E (2008) The Phasor Approach to Fluorescence Lifetime Imaging Analysis. Biophysical journal 94: L14–L16. 17981902 10.1529/biophysj.107.120154 PMC2157251

[52] Ausubel FM, Brent R, Kingston RE, Moore DD, Seidman JG, et al. (1987–2014) Current Protocols in Molecular Biology. John Wiley and sons Vol 1–5.

[53] Ai H, Henderson JN, Remington SJ, Campbell RE (2006) Directed evolution of a monomeric, bright and photostable version of Clavularia cyan fluorescent protein: structural characterization and applications in fluorescence imaging. Biochem J 400: 531–540. 16859491 10.1042/BJ20060874 PMC1698604

[54] Griesbeck O, Baird GS, Campbell RE, Zachariast DA, Tsien RY (2001) Reducing the environmental sensitivity of yellow fluorescent protein. J Bio Chem 276: 29188–29194. 11387331 10.1074/jbc.M102815200

[55] Murakoshi H, Lee SJ, Yasuda R (2008) Highly sensitive and quantitative FRET-FLIM imaging in single dendritic spines using improved non-radiative YFP. Brain Cell Biol 36: 31–42. 18512154 10.1007/s11068-008-9024-9 PMC2673728

[56] Colicelli J, Birchmeier C, Michaeli T, O'Neill K, Riggs, et al (1989) Isolation and characterization of a mammalian gene encoding a high-affinity CAMP phosphodiesterase. Proc Natl Acad Sci USA 86: 3599–3603. 2542941 10.1073/pnas.86.10.3599 PMC287185

[57] Ammerer G (1983) Expression of genes in yeast using the ADCI promoter. In: Recombinant DNA Part C, vol. 101: Elsevier (Methods in Enzymology), pp.192–201. 10.1016/0076-6879(83)01014-9 6310322

[58] Tong AH, Evangelista M, Parsons AB, Xu H, Bader GD, et al (2001) Systematic genetic analysis with ordered arrays of yeast deletion mutants. Science 29: 2364–2368. 10.1126/science.1065810 11743205

[59] Roman H (1956) A system selective for mutations affecting the synthesis of adenine in yeast. Compt Trav Lab Carlsberg Ser Physiol 26: 299–314.

[60] Covey TM, Vira MA, Westfall M, Gulrajani M, Cholankeril M, et al (2013) Single cell network profiling assay in bladder cancer. Cytometry A 83: 386–95. 23300058 10.1002/cyto.a.22244

[61] Bodenmiller B, Zunder ER, Finck R, Chen TJ, Savig ES, et al (2012) Multiplexed mass cytometry profiling of cellular states perturbed by small-molecule regulators. Nature Biotechnol 30: 858–867. 22902532 10.1038/nbt.2317 PMC3627543

[62] Bendall SC, Davis KL, Amir el-AD, Tadmor MD, Simonds EF, et al (2014) Single-cell trajectory detection uncovers progression and regulatory coordination in human B cell development. Cell 157: 714–725. 24766814 10.1016/j.cell.2014.04.005 PMC4045247

[63] Colman-Lerner A, Gordon A, Serra E, Chin T, Resnekov O, et al (2005) Regulated cell-to-cell variation in a cell fate decision system. Nature 437: 699–706. 16170311 10.1038/nature03998

[64] Gohar AV, Cao R, Jenkins P, Li W, Houston JP, et al (2013) Subcellular localization-dependent changes in EGFP fluorescence lifetime measured by time-resolved flow cytometry. Biomedical Optics Express 4: 1390–1400. 24010001 10.1364/BOE.4.001390 PMC3756581

[65] Sheff MA, Thorn KS (2004) Optimized cassettes for fluorescent protein tagging in Saccharomyces cerevisiae. Yeast 21: 661–670. 15197731 10.1002/yea.1130

[66] Frigo M, Johnson SG (2005) The Design and Implementation of FFTW3. Proceedings of the IEEE 93: 216–231.

[67] Beck R, Dempster AG, Kale I (2001) Finite-precision Goertzel filters used for signal tone detection. In *IEEE Trans. Circuits Syst.**II* 48: 691–700.

