# Supplementary figures and images for: Measuring and Sorting Cell Populations Expressing Isospectral Fluorescent Proteins with Different Fluorescence Lifetimes
Source: PLoS One. 2014 Oct 10;9(10):e109940. doi: 10.1371/journal.pone.0109940 (PMC4193854; doi:10.1371/journal.pone.0109940)

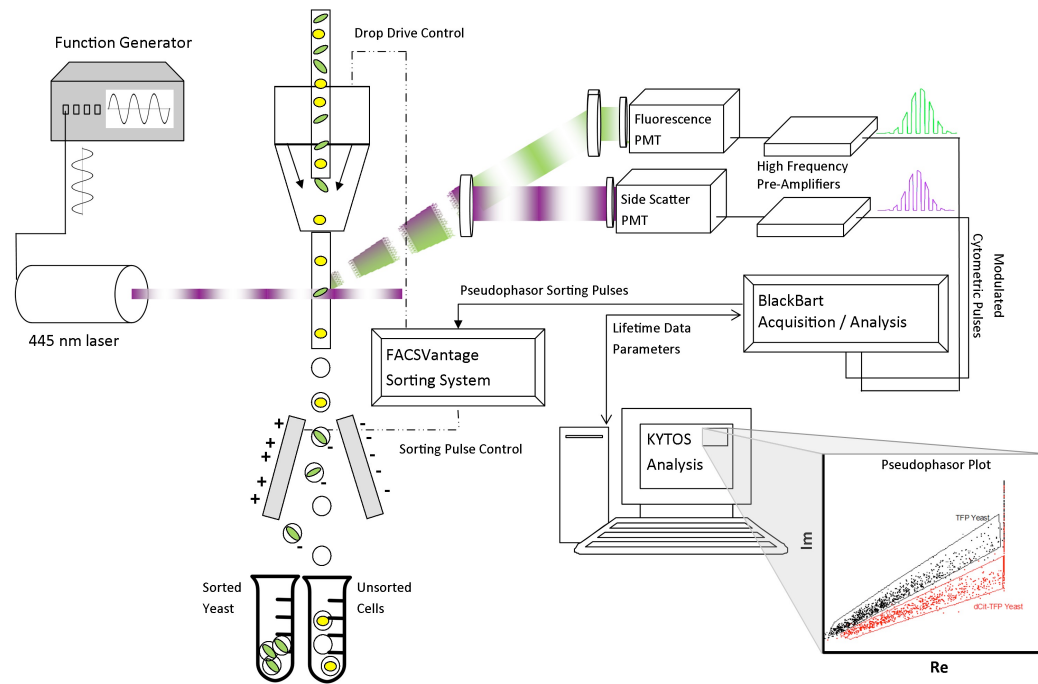

Supplement: Figure S1 — Diagram of the modified cytometer. Simplified diagram of the flow cytometric lifetime detection/sorting system. Yeast expressing fluorescent proteins rapidly transit a modulated laser beam from a diode laser, driven by a sine wave from a function generator at RF frequencies, following hydrodynamic focusing within the cytometer nozzle. Scattered laser light and fluorescence are collected 90° incident to the laser excitation beam and are optically focused, split, and filtered onto photomultiplier tube (PMT) detectors. The resultant photocurrent signal is amplified utilizing high frequency PMT preamplifiers that do not attenuate the modulated Gaussian shaped signal. These signals are sampled by the BlackBart data acquisition/analysis system, which relays cytometric information (pulse height, width) as well as pulse waveforms to a host computer running KYTOS analysis software. From KYTOS the phase shift between the two signals, the fluorescence lifetime, and pseudophasor information can be analyzed and recorded. Sort gates can also be set within the pseudophasor plot which are relayed back to the BlackBart system, where an FPGA based analysis analogous to that happening on KYTOS will be performed. When events occur with pseudophasor parameters that fall within the predefined sort gates, sorting pulses are relayed to the FACSVantage sorting system. The FACSVantage drives the drop drive control in which a piezoelectric device oscillates at a specific frequency and imparts vibration into the fluid stream that causes droplets to form. When an event that falls within the sorting gates is detected the FACSVantage sorting system will charge the fluidic stream right before the moment of droplet breakoff, leaving the resultant drop with an electrical charge. As the charged droplet containing the particle of interest falls, it passes through two strongly charged deflection plates. The charged particle will be attracted/deflected one way depending on its charge. These cells of int [file pone.0109940.s001.pdf]

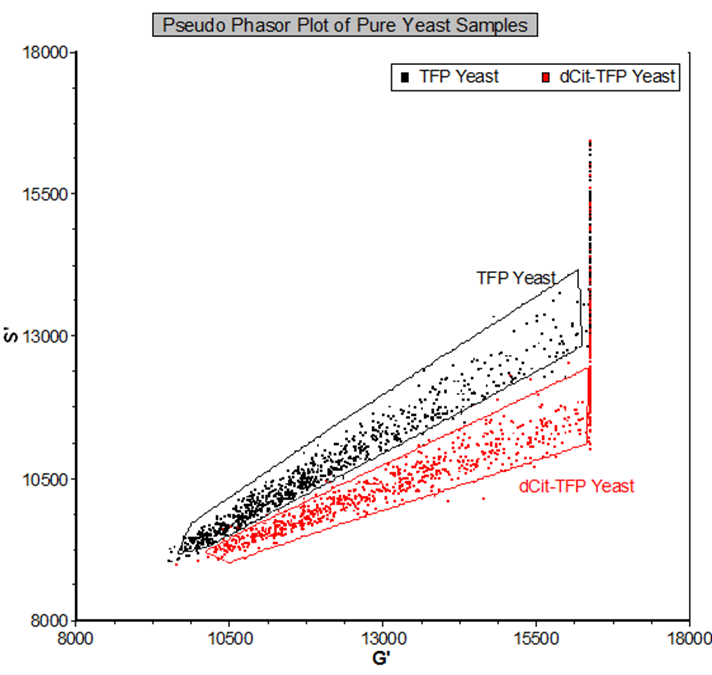

Supplement: Figure S2 — Pseudophasor sort gates. Screen capture of a pseudophasor plot produced on the FACSVantage, with operator-drawn polygonal sort gates. (TIF) [file pone.0109940.s002.tif]

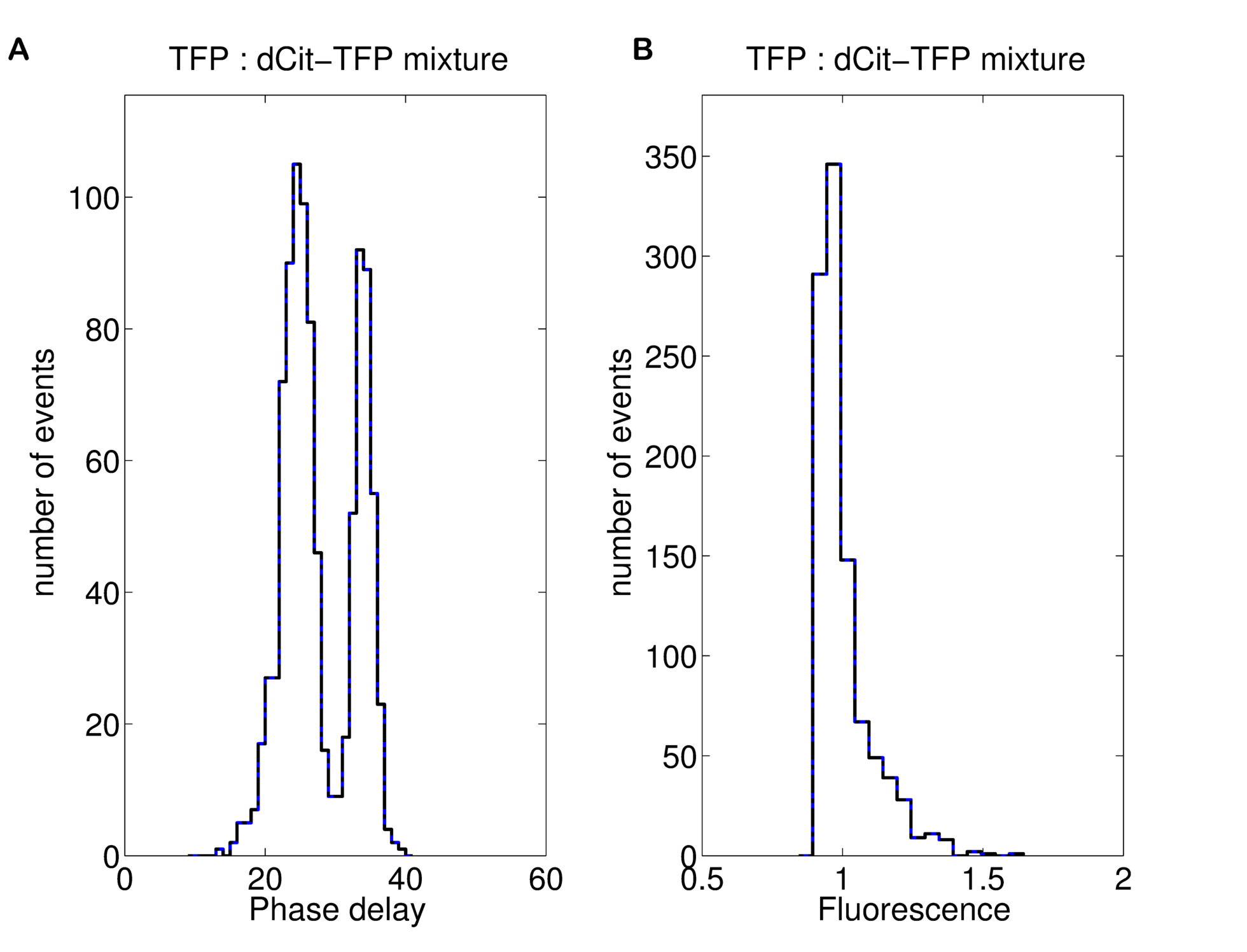

Supplement: Figure S3 — Histograms from mixed cell populations. Data from 1000 events, for a mixture of cells expressing TFP and cells expressing dCit-TFP. A) Phase delay of fluorescence signal in degrees, showing two clear peaks. B) Fluorescence intensity in arbitrary units, showing only one peak. (TIF) [file pone.0109940.s003.tif]

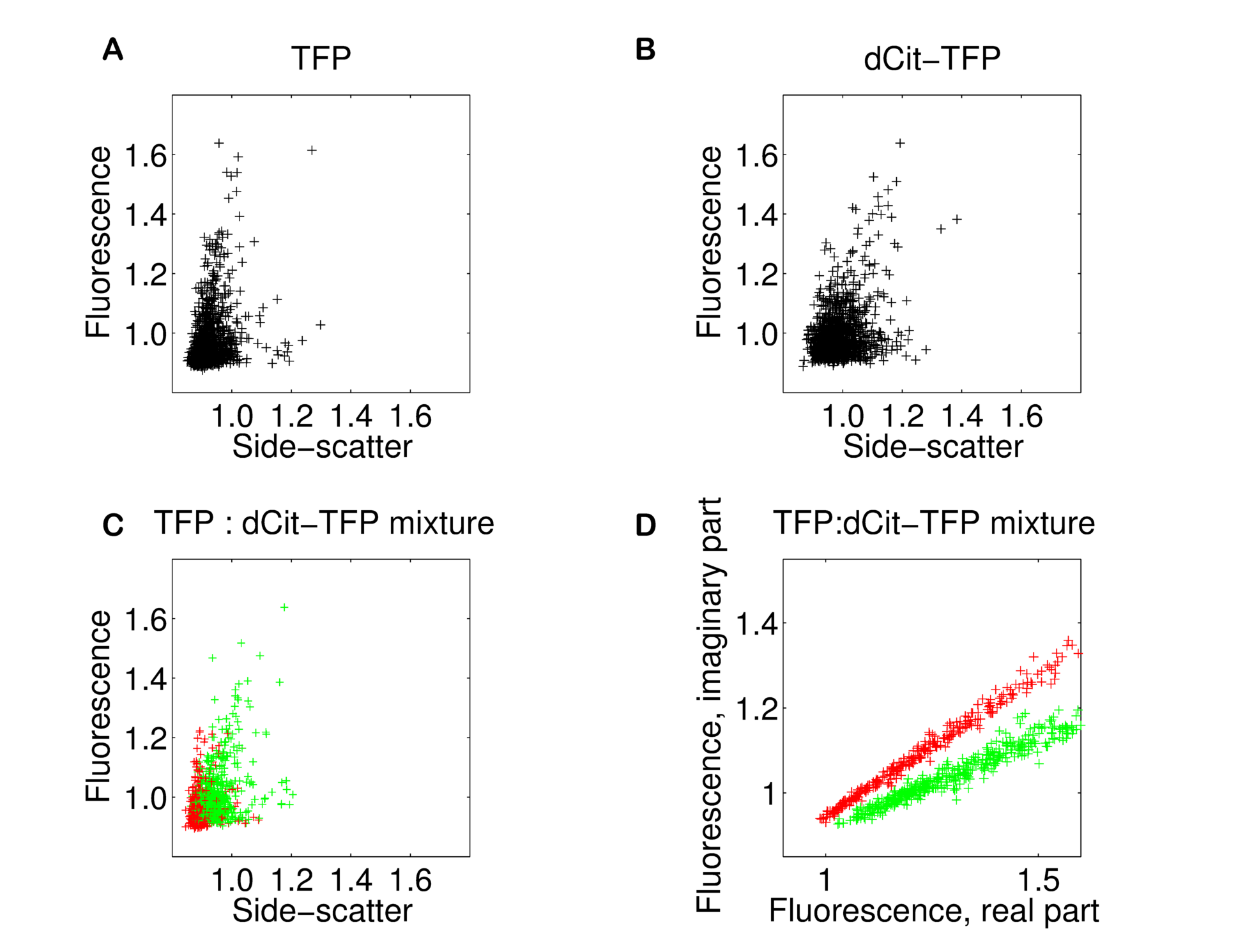

Supplement: Figure S4 — Detecting lifetime differences in flow. A) Intensity scatter plot for the BSY034 TFP strain. B) Intensity scatter plot for the BSY035, the dCit-TFP strain. C) Intensity scatter plot for mixture of BSY034 and BSY035, colored according to phase. Green dots represent phase delays of less than 30 degrees, red dots, phase delays more than 30 degrees. D) Pseudophasor plot of the data from 3C. (TIF) [file pone.0109940.s004.tif]

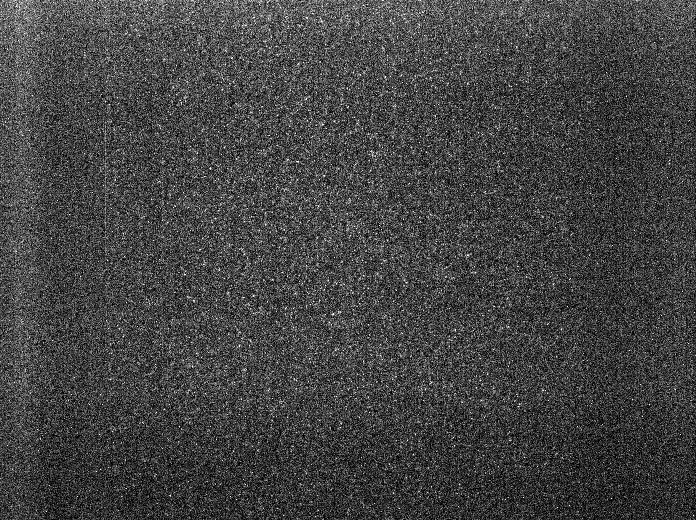

Supplement: Figure S13 — Cit.zip: FLIM data archive with TIFF phase stacks of BSY004 cells, reference phase stacks of Erythrosin-B solution, and data format documentation. (ZIP) [file pone.0109940.s013.zip › Reference 75ms_1.tif]

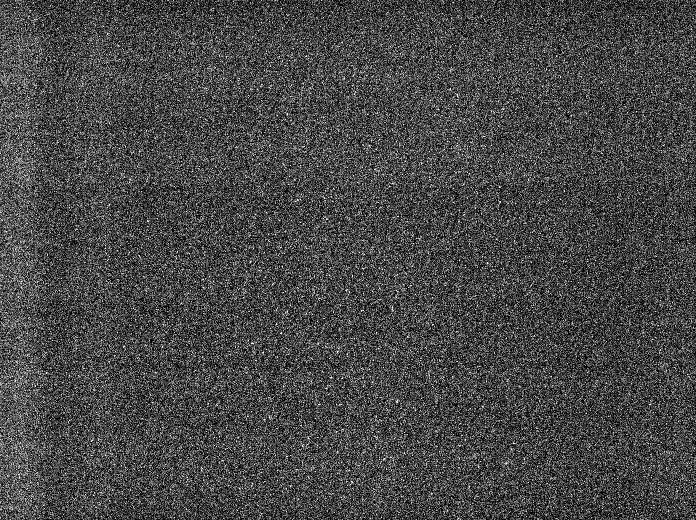

Supplement: Figure S13 — Cit.zip: FLIM data archive with TIFF phase stacks of BSY004 cells, reference phase stacks of Erythrosin-B solution, and data format documentation. (ZIP) [file pone.0109940.s013.zip › Sample citrine 10ms_1.tif]
