# Supplementary material for: Measuring and Sorting Cell Populations Expressing Isospectral Fluorescent Proteins with Different Fluorescence Lifetimes
Source: PLoS One. 2014 Oct 10;9(10):e109940. doi: 10.1371/journal.pone.0109940 (PMC4193854; doi:10.1371/journal.pone.0109940)
Supplement: Table S1 — Primer Sequences. Primers used to engineer XFP constructs. (PDF) [file pone.0109940.s015.pdf]

Primer	Sequence
BS1	AAGCTTATGTCTAAAGGTGAAGAATTATTCAC
BS2	GCGGCCGCTTATTTGTACAATTCATCCATACCAT
BS3	GCTACTTACGGTAAATTGACCTTAAAATTGATTTGTACTACTGGTAAATTGCC
BS4	CATTACTTATCCTATCAATCTAAGTTATCCAAAGATCCAAACGAAAAG
BS5	GGTCACAAATTGGAATACAACTGGAACTCTCACAATGTTTACATC
BS6	GACCACATGGTCTTGTTAGAAAGAGTTACTGCTGCTGGTATTACCCATG
BS7	CTCCAAGCTTATGGTGTCCAAAGGCG
BS8	GGCCGCGGCCGCTTATTTGTAGAGTTCGTCCATACC
BS9	GTCGTAAGCTTATGTCTAAAGGTGAAGAATTATTCAC
BS10	GTCGAAGCTTACCACCACTTCCACCTTTGTACAATTCATCCATACCATGGGTA

Restriction sites are underlined
